# Tumors vs. Chronic Wounds: An Immune Cell's Perspective

**DOI:** 10.3389/fimmu.2019.02178

**Published:** 2019-09-12

**Authors:** Yichao Hua, Gabriele Bergers

**Affiliations:** ^1^Laboratory of Tumor Microenvironment and Therapeutic Resistance, Department of Oncology, VIB-Center for Cancer Biology, KU Leuven, Leuven, Belgium; ^2^Department of Neurological Surgery, UCSF Comprehensive Cancer Center, UCSF, San Francisco, CA, United States

**Keywords:** myeloid cells, macrophages, neutrophils, endothelial cells, tumor vessels, wound repair, antiangiogenic immunotherapy

## Abstract

The wound repair program is tightly regulated and coordinated among different cell constituents including epithelial cells, fibroblasts, immune cells and endothelial cells following consecutive steps to ensure timely, and proper wound closure. Specifically, innate and adaptive immune cells are pivotal participants that also closely interact with the vasculature. Tumors are portrayed as wounds that do not heal because they undergo continuous stromal remodeling and vascular growth with immunosuppressive features to ensure tumor propagation; a stage that is reminiscent of the proliferative resolution phase in wound repair. There is increasing evidence from mouse model systems and clinical trials that targeting both the immune and vascular compartments is an attractive therapeutic approach to reawaken the inflammatory status in the “tumor wound” with the final goal to abrogate tumor cells and invigorate tissue homeostasis. In this review, we compare the implication of immune cells and the vasculature in chronic wounds and tumor wounds to underscore the conceptual idea of transitioning tumors into an inflammatory wound-like state with antiangiogenic immunotherapies to improve beneficial effects in cancer patients.

## Introduction

Neoplastic conversion of cells into a malignant tumor with metastatic properties acquires not only multiple intrinsic traits but also necessitates the participation of the tumor microenvironment with its diverse cellular and matrix constituents (1). Notably, innate immune cells, and specifically macrophages, are functionally involved in nearly every stage of the multistep cascade of tumorigenesis (2). There is also increasing evidence that neutrophils functionally contribute to distinct stages, which includes angiogenesis, escape of tumor dormancy, and metastatic seeding (3, 4). Of the many cancer hallmarks, the onset of tumor neovascularization, and escape of immunosurveillance are two environmental traits that are codependent. They encompass endothelial and mural cells constituting the vasculature as well as innate and adaptive immune cells that partake in heterotypic interactions with one another (5). This crosstalk is not tumor-specific but attributed to their traditional roles in tissue repair where immune cells also affect vascular properties while endothelial cells direct immune cell trafficking and survival.

## Immune Cells in Wound Healing

**Figure 1:**
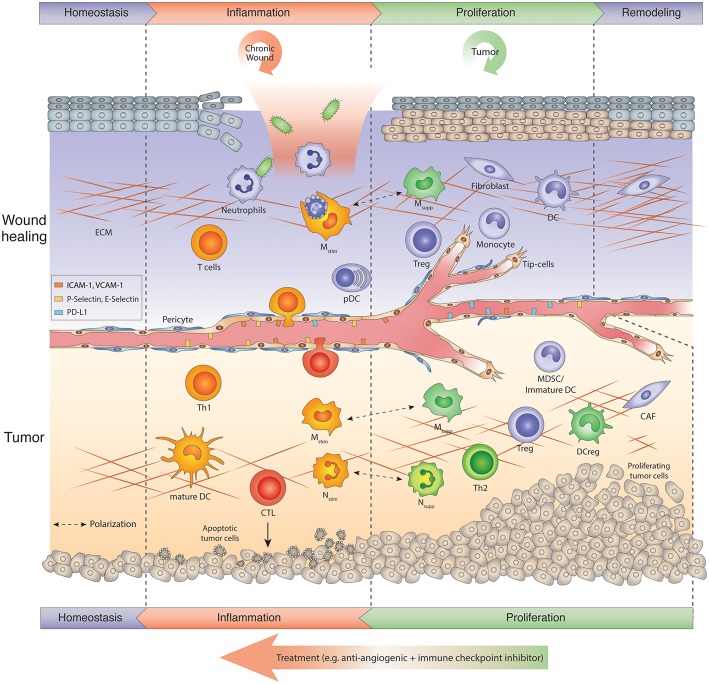
Tumors hijack the wound repair program: Chronic wound vs. tumor wound. Usually wound healing is manifested in several sequential steps after injury referred to as inflammation, proliferation-resolution, and remodeling phase. Immune cells are key regulators in the wound repair program. In the inflammation phase, neutrophils kill microbes and macrophages phagocytose apoptosing neutrophils, while skin-resident or infiltrating T cells produce IL-17, IL-22, and TNF α to amplify the host defense response. During the proliferation phase, macrophages switch to an anti-inflammatory phenotype (M_supp_). M_supp_ macrophages, N_supp_ neutrophils (or tumor-associated neutrophil, TANs), Tregs and other immunosuppressive cells may help to attenuate the inflammation response and facilitate resolution and tissue remodeling. Chronic wounds get trapped in the inflammation phase, exacerbate inflammation and thus, hinder tissue repair. Tumors, on the other hand, hijack the proliferative/remodeling program and provide signals that create a continuous angiogenic and immunosuppressive environment enabling tumors to grow and escape immune surveillance. Therefore, tumors remain in the proliferative phase upon the onset of angiogenesis. Antiangiogenic immunotherapies induce an inflammation program in tumors that reawakens and boosts an anti-tumor response. The ultimate goal is to create a homeostatic situation in which tumor cells are eliminated and a normal tissue architecture is achieved. CAF, Cancer-associated fibroblast; CTL, cytotoxic T lymphocyte; DC, dendritic cell; DCreg, regulatory DC; ECM, extracellular matrix; MDSC, myeloid-derived suppressor cells; Mono, monocyte; M_stim_, immunostimulatory macrophage (M1-like); M_supp_, immunosuppressive macrophage (M2-like); N_stim_, immunostimulatory neutrophil (N1-like); N_supp_, immunosuppressive neutrophil (TAN); pDC, plasmacytoid DC; Th, T helper cell; Treg, regulatory T cell.

Acute wound healing, being extensively studied in the skin and gut, follows a well-coordinated multistep process that constitutes inflammation, proliferation and remodeling phases to restore tissue homeostasis, regain function, and protect from infection (6–9) (Figure 1, upper panel). Following immediate hemostasis to impede bleeding, and as a first defense mechanism, neutrophils, and then CCR2^+^ monocytes and macrophages are recruited to the wound and activated by proinflammatory cytokines (e.g., TNFα, IL1) and chemokines (e.g., CXCL-1,5,8; CCL-2) -secreting epithelial cells and fibroblasts and cellular contents (e.g., DNA, RNA, uric acid, metabolites, HMGB1) from dying cells that serve as danger signals (DAMPs) (10, 11). During this inflammation period, neutrophils secrete reactive oxygen species (ROS), nitric oxide (NO), and antimicrobial proteins (AMPs) and deploy web-like extracellular traps (NETs) in order to phagocytose and kill contaminating microorganisms (12, 13). Neutrophils also produce TNFα, IL1β, IL-6, CXCL2/8 as well as MCP-1 (monocyte attracting protein-1) that recruit macrophages, T cells as well as additional neutrophils to the wound thus amplifying a Th1 proinflammatory response. Inflammatory macrophages predominantly serve as scavengers removing dead cells and cellular debris. They also produce similar cytokines, including IL-12/23 as well as IFNγ that recruit T-cells and natural killer cells (NK), and stimulate their proinflammatory responses (14, 15). In addition, endothelial cells in dermal venules upregulate the lymphocyte adhesion molecules V-CAM-1, I-CAM-1, E- and P-selectins, which regulate lymphocyte rolling and tethering, and thus augment lymphocyte infiltration into the wound (7, 16). Consequently, T cells in the wound produce interleukin (IL)-17, IL-22, and tumor necrosis factor a (TNFα), which further intensifies the defense response of the immune system (Figure 1, upper panel). In addition, plasmacytoid dendritic cells (pDC) infiltrate the wound and recognize nucleic acids from injured cells leading to the production of type I interferons (17). Further, dermal conventional dendritic cell type 1 (cDC1s) can cross-present antigens (6, 18, 19) to facilitate T cell function, and control the generation of commensal-specific CD8^+^ IL-17^+^ T cells in the skin (20). As soon as neutrophils complete their mission, they undergo apoptosis and are removed by macrophages (21). This phagocytotic activity instigates the transition to an anti-inflammatory Th2-like phenotype in macrophages and ends the inflammatory period (21). The conversion from a “Th1” to “Th2” state is indeed an essential and critical step to impede inflammation and necessary to initiate the proliferative and resolution phase for efficient wound repair (Figure 1, upper panel) (22). If the wound repair cannot proceed beyond the inflammation phase, it will generate a chronic wound with barrier defects (8, 9, 23). During the proliferative resolution phase, granulation tissue fills the wound with connective tissue, and keratinocytes, fibroblasts, and endothelial cells expand to enable a proper wound closure. Therefore, anti-inflammatory Th2-like “repair” macrophages activate fibroblasts that in turn incite keratinocyte proliferation and migration and together promote neovascularization by directly secreting Vascular Endothelial Growth Factor (VEGF), Transforming Growth Factor β1 (TGF-β1), and IL8 as well as other factors including metalloproteinases (24). During wound healing, the generation of a new vascular network is predominantly caused by sprouting by which new vessel growth is initiated from activated preexisting capillary endothelial cells. In addition, but to a much lesser extent, bone marrow-derived hematopoietic precursors, and even dendritic cells and monocytes, can also be recruited to the growing vasculature where they differentiate into endothelial cells (25–29).

Expanding vascular sprouts exist of proliferating endothelial stalk cells and migrating tip cells at the leading edge which follow a gradient of proangiogenic factors produced by various cells including keratinocytes and stromal cells. Tip cells of different sprouts connect by anastomosis under the chaperon of macrophages, followed by maturation of the new vessel to enable blood flow (30). The entire process is tightly regulated by several proangiogenic factors (e.g., VEGF, PIGF, FGF, IL8) as well as antiangiogenic factors (e.g., Sprouty2, pigment epithelium-derived factor (PEDF), CXCL10) displaying a fine balance of both vascular growth and remodeling until vessels become covered with pericytes, form a basement membrane and mature (24, 31, 32). Although the implication of macrophages has been well-established in the distinct steps of wound healing, the role of neutrophils in the later stages, specifically in angiogenesis has not been appreciated until recently. Like macrophages, neutrophils can polarize from an immunostimulating N1 phenotype to an immunosuppressive N2 status in which they, like macrophages, produce VEGF and MMPs and other angiogenic factors (3, 33). For example, neutrophil-produced VEGF appears to be crucial in the healing process of an injured cornea in mice because antibody-mediated neutrophil depletion substantially impaired neovascularization (34). Also, dendritic cell expansion in the skin can enhance wound healing by DC-produced factors that promote re-epithelialization, angiogenesis, granulation tissue formation, growth factor production (35). Finally, during the last phase of wound repair, the immune cell composition reverses back to normal levels, and the extracellular matrix in the wound undergoes further remodeling to properly close the wound, a process that can persist for weeks to months (8, 9).

## Tumors are Non-HEALING Wounds But Differ From Chronic Wounds

While the acute wound healing cascade is tightly regulated and coordinated, chronic wounds (like in diabetes or ulcers) develop when the repair process is trapped, most commonly in the inflammatory response phase being unable to trigger the repair program in macrophages to move to the next phase. Consequently, an excessive immune response develops that leads to further tissue damage rather than tissue restoration (23). In the late 80s, Harold Dvorak compared tumors to wounds that never heal (36). The difference to chronic wounds, however, is that “tumor wounds” avoid the inflammatory phase to escape immunosurveillance, and hijack the proliferative resolution program of the wound repair to induce a vascular-rich stroma with immunosuppressive and angiogenesis-promoting cell constituents conducive to tumor propagation (Figure 1) (36). Similar to the processes in the resolution phase of wounds, tumors instigate several remodeling processes that include increased vascular permeability, the onset of angiogenesis and deposition of an extravascular fibrin-enriched provisional stroma which is replaced by a vascular connective granulation tissue causing desmoplasia in certain tumor types (37). Concomitantly, tumors polarize innate immune cells from an immunostimulating to an immunosuppressive and angiogenic state and thus, not only escape immunosurveillance but also take advantage of myeloid-produced angiogenic factors that help to expand its tumor vasculature accommodating the needs of a growing tumor (Figure 1, lower panel) (38). Notably, the process of angiogenesis in wounds and tumors is regulated by similar factors, but in contrast to the tight regulation of angiogenesis in acute wounds, the production of angiogenesis-promoting and inhibiting molecules in tumors is imbalanced (39, 40). Tumors continue to stimulate neovascularization, which results in an expanding tumor vasculature with an abnormal phenotype displaying hyperdilated tumor vessels with poor pericyte coverage and leaky and sluggish blood flow (41). Subsequently, a hypoxic and acidic environment in tumors with increased interstitial pressure evolves that further elevates the production of proangiogenic factors and thus exacerbates a proangiogenic response (40, 42).

## Innate Immune Cells Promote Angiogenesis

Like in wounds, myeloid cells present a prominent population in tumors where they can make up to 30% of the entire population dependent on tumor type and stage (5, 43–45). As soon as myeloid cells reach the tumor, some of the immature innate immune cells will differentiate into tumor-associated macrophages (TAMs) and neutrophils while others remain in an immature stage resembling monocytic myeloid-derived suppressor cells (M-MDSCs) and immature DCs or granulocytic MDSCs (G-MDSCs) (46). In addition, the presence of regulatory (reg) DCs has also been described which suppress T cell activation and proliferation and enable Treg differentiation and expansion (47–49).

Importantly, however, the cytokine milieu to which myeloid cells are exposed, and the specific tumor microenvironment in which they reside will dictate which phenotype these plastic cells will display. IFNγ, TNFα, and IL-12 promote an immunostimulatory polarization in innate immune cells while TGF-β, IL-4, IL-10, and CSF-1 are prominent factors that skew macrophages and neutrophils toward an immunosuppressive and angiogenic phenotype, and promote Treg proliferation (3, 50, 51).

**Figure 2:**
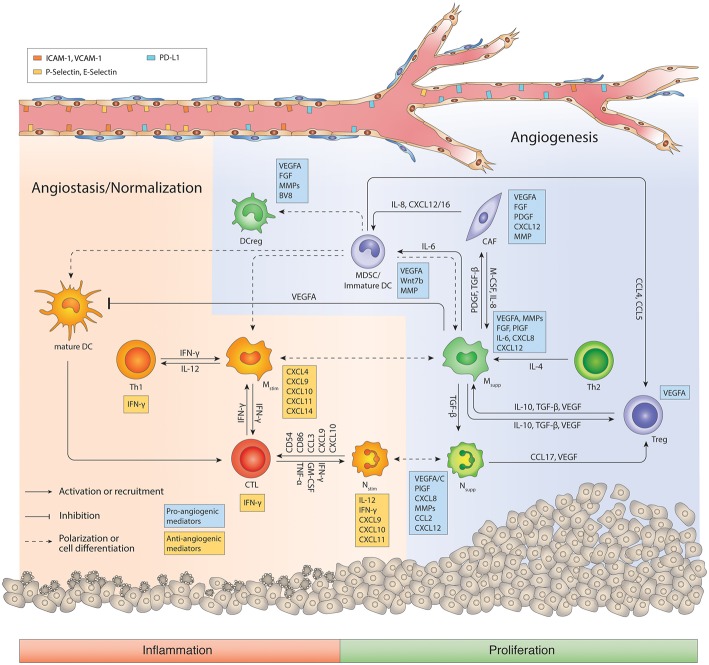
Regulatory network of the tumor immune microenvironment. The tumor microenvironment facilitates cross-talks between immunosuppressive macrophages, MDSCs, N_supp_, Tregs and CD4^+^ Th2 cells that promotes angiogenesis, immunosuppression, and tumor progression. During the course of antiangiogenic and/or immunotherapy, myeloid cells including macrophages can switch to an inflammatory phenotype, and cooperate with CD8^+^ CTLs and CD4^+^ Th1 cells to generate an anti-tumor response and promote vessel pruning and normalization. Ang2, angiopoietin 2; CAF, Cancer-associated fibroblast; CTL, cytotoxic T lymphocyte; DC, dendritic cell; DCreg, regulatory DC; FGF, Fibroblast growth factor; IFN, interferon; IL, interleukin; M-CSF, Macrophage colony-stimulating factor; MDSC, myeloid-derived suppressor cells; MMP, Matrix metalloproteinases; M_stim_, immunostimulatory macrophage (M1-like); M_supp_, immunosuppressive macrophage (M2-like); N_stim_, immunostimulatory neutrophil (N1-like); N_supp_, immunosuppressive neutrophil (TAN); PDGF, Platelet-derived growth factor; PlGF, Placental growth factor; TAN, Tumor-associated neutrophil; TGF-β, Transforming growth factor beta; Th, T helper cell; Treg, regulatory T cell; VEGFA, Vascular endothelial growth factor A. Cells in orange or red color represent immunostimulation/type 1 immunity/anti-angiogenic status, while cells in green/blue represent immunosuppression/type 2 immunity/pro-angiogenic status.

Thus, although macrophages and neutrophils have the ability to inhibit angiogenesis and attack whatever they consider foreign, in tumors, they commonly promote an escape of immunosurveillance, and new vessel formation. There is strong evidence of the functional significance of TAMs in tumor angiogenesis in multiple systems. One of the first seminal studies demonstrating the relevance of TAM-directed tumor angiogenesis was achieved in the mammary virus-polyoma middle T- antigen (PyMT) breast tumor model, and then confirmed in other tumor model systems (52–54). Thereby, macrophages were depleted in tumors by genetically or pharmacologically impairing CSF1-CSF1R signaling, which is essential for macrophage differentiation and survival, or by broad elimination of myeloid cells with clodronate liposomes. As a result, macrophage-deficiency in these various murine tumor models reduced angiogenic activity (52, 54–56). Again, however, the pro-angiogenic capacity of TAMs is dependent on the cytokine milieu, which in part is triggered by a hypoxic and acidic microenvironment (40, 57). These conditions induce the secretion of chemotactic factors such as VEGF, colony-stimulating factor 1-3 (CSF1-3), the CX chemokines CXCL12 (aka SDF1a) and CX3CL1, the CC-chemokines CCL2, CCL5, CCL22, interleukin IL6, semaphoring 3A and others that recruit immune cells to the tumor where they become programmed to facilitate angiogenesis by secreting proangiogenic factors (40, 43, 46, 54, 58–63) (Figure 2). In this pro-tumor state, myeloid cells represent a crucial source of angiogenic factors producing VEGF, fibroblast growth factor 2 (FGF2), CXCL8 (CXC-chemokine ligand 8), WNT7B, and BV8. In addition, they produce PDGF-B, PIGF, Neuropilin-1, IL-6, and several proteinases, including matrix metalloproteinases (MMPs) and cathepsins, which also have pro-angiogenic properties (64, 65) (Figure 2). Certainly, hypoxia via HIF-1α also augments the secretion of proangiogenic cytokines in tumor cells, specifically VEGF, which is the most prominent angiogenic factor being highly expressed in a variety of different tumor types (66). This makes tumor cells the major source of VEGF and raises the question as to why myeloid cells also induce VEGF in response to hypoxia (67–70). As it is well-established that VEGF contributes to tumorigenesis (71), Stockmann et al. made a surprising observation that myeloid cell-specific VEGF deletion in mice enhanced the development of spontaneous mammary PYMT tumors and tumors of several subcutaneous isograft models (53). Interestingly, VEGF depletion in macrophages promoted tumor vessel normalization and thus enhanced the exposure of tumors to chemotherapeutic cytotoxicity (53). This is an important study that supports the notion that not only total VEGF levels but also the location of VEGF within the tumor regulate vascular characteristics. It appears that likely perivascular macrophages secrete VEGF to fine-tune angiogenic properties of blood vessels by closely interacting with endothelial cells. Congruent with these observation of location-dependent effects of VEGF, myeloid cell-produced VEGF has also been shown to promote the intravasation of tumor cells into the blood stream by enhancing vascular permeability (72). Besides producing VEGF, myeloid cells regulate VEGF bioavailability by releasing matrix metalloproteinase MMP-9 to liberate sequestered VEGF from the extracellular matrix. This enables VEGF binding to and activation of VEGFR2 on endothelial cells at sites of neovascularization (59, 73) (Figure 2). Another example of location-dependent regulation and function of TAM activity has been described for semaphorin 3A (Sema 3A). Sema 3A is induced by hypoxia and was found to recruit macrophages by binding to neuropilin-1 (Nrp-1) and PlexinA1/A4 co-receptors and signaling through VEGFR1. As soon as macrophages localized in low-oxygen conditions, expression of Nrp-1, but not PlexinA1/A4, was repressed in macrophages, which trapped macrophages in these hypoxic areas where they facilitated angiogenic and immunosuppressive properties (63). Congruently, genetic deletion of Nrp-1 in macrophages was sufficient to impair TAM recruitment and accumulation in hypoxic regions, resulting in impaired neovascularization, improved antitumor immunity and consequently, delayed tumor growth (63). TIE2-expressing macrophages (TEMs) have highly angiogenic characteristics which, like TAMs, correlate with vascular density in various murine and human tumors (74, 75). TEMs are preferentially found in close association with blood vessels being recruited by angiopoietin 2 (ANGPT2)-secreting endothelial cells. ANGPT2 promotes angiogenesis in an autocrine manner by binding to the TIE2 receptor on endothelial cells and mediates interactions between endothelial cells and TEMs to support vessel sprouting and macrophage -directed anastomosis (30, 46, 76). Albeit TEMs compose a minor subset of TAMs, they have been found to be highly relevant in promoting tumor angiogenesis because TEM depletion experiments using antibody-mediated neutralization of the Tie2 ligand Ang2 or Tie2 promoter-driven thymidine kinase both reduced angiogenesis and tumor propagation in mammary, pancreatic neuroendocrine and brain tumor mouse models (76, 77). Besides macrophages, neutrophils have now also been recognized to be important mediators of tumorigenesis but the TAN-dependent mechanisms of tumor progression are not fully understood (3, 4, 78). G-CSF mediates neutrophil proliferation and differentiation by binding to CSF3R and activating downstream Janus kinase (JAK)–signal transducer and activator of transcription 3 (STAT3) pathways. CXCL chemokines including CXCL8 as well as IL-1β, IL17, and IL-6 predominantly mediate the recruitment of neutrophils to tumors (3, 79–81) (Figure 2). In contrast to injuries, where neutrophils are the first cells to enter the wound to fight contaminants and then undergo efferocytosis, neutrophils in tumors (TAN) do not appear to apoptose but like macrophages become polarized to an immunosuppressive and angiogenic phenotype. These observations of phenotypic neutrophil modulation have led to the notion that the functional plasticity seen in other immune cells, such as TAMs, may also be reflected in TANs (3). In support, cytokine-driven polarization of neutrophils in murine models of cancer have provided evidence that the cytokine TGF-β and type I interferons are key effectors of neutrophil polarization. TGF-β skews neutrophils toward an N2 phenotype. It blocks neutrophil production of ROS, reactive nitrogen intermediates, and IL-1β and impedes neutrophil degranulation in response to LPS. Conversely, TGF-β inhibition or the presence of type I interferons polarize neutrophils to an N1 phenotype while inhibiting type I interferon signaling unleashes N2 properties in neutrophils (82). N2 conversion, similar to M2 macrophage polarization, may in part be caused by hypoxia, which has been shown to delay neutrophil apoptosis (83). Mechanistically, hypoxia induced neutrophil survival through HIF-1α-dependent NF-κB activity under low-oxygen tension in a PHD3-dependent manner (57, 84). Like TAMs, TANs produce similar proangiogenic factors and proteases like VEGF, FGF, BV8, and MMP9, which is in part regulated by STAT3 signaling (81, 85–87). The angiogenic expression profile appears to be very conserved because in zebrafish, transcriptomic profiling of liver tumor-associated neutrophils revealed up-regulation of similar gene transcripts promoting angiogenesis (88). VEGF is the prominent angiogenic factor that neutrophils, like TAMs, not only express and secrete but they also carry it in granules which are released upon TNF stimulation (89). TANs, like TAMs, provide another quick route of VEGF accessibility to activate endothelial cells by releasing MMP-9 to release sequestered VEGF from the extracellular matrix (ECM) (90, 91). Indeed, this neutrophil-dependent mechanism was critical to instigate the angiogenic switch in the dysplastic stage of pancreatic islets in the Rip1Tag2 endogenous pancreatic neuroendocrine tumor model because not only MMP-9 inhibition but also neutrophil depletion was sufficient to diminish the angiogenic switch (73, 90). Further, GM-CSF stimulated tumor-associated neutrophils to produce the angiogenic factor Bv8 in several murine tumor models, which in turn attracted more neutrophils, thus, providing a forward loop for neutrophil recruitment and activation (92). Consequently, pharmacological or genetic blockade of CSF3, CSF3R, or BV8 decreased the number of TANs and inhibited tumor angiogenesis and growth (81). It is notable that in addition to the identification of intratumoral neutrophils, three distinct neutrophil populations have recently been described in the blood circulation, both in mice and in patients with advanced cancer (93). High- density neutrophils are reminiscent of cancer-killing N1 neutrophils while mature LDNs are not cytotoxic and display impaired functionality and immunosuppressive properties. The third population consists of morphologically immature LDNs which show characteristics of granulocytic myeloid-derived immunosuppressive cells (MDSCs). They are also observed in tumors, and thus suggest the other circulating neutrophil populations may be present in tumors as well (93). MDSCs are immature myeloid cells of granulocytic (G-MDSC) or monocytic (M-MDSC) origin, first discovered in tumors, that not only strongly suppress CD4 and CD8 T cells but also convey angiogenic features (43, 94, 95). MDSCs, as well as reg-DCs, secrete proangiogenic factors similar to M2-like TAMs and N2-like TANs, such as VEGF, FGF2, BV8, and MMP9 (79). Tumor -produced CSF3, IL-1β, and IL-6 activate STAT3 in MDSCs which leads to their expansion but hinders MDSC maturation into macrophages or neutrophils. Notably, the proangiogenic expression profile of MDSCs conceivably overlaps with those of TAMs and TANs (85, 87, 94). Indeed, it has become apparent from several studies that the different innate immune cell populations produce several but similar angiogenic molecules to facilitate neovascularization. Given the functional redundancy in their angiogenic properties, it is conceivable that myeloid cells can compensate for the lack of other myeloid cell constituents to regulate tumor angiogenesis. Indeed, neutrophils can compensate for macrophages to support tumor angiogenesis in tumor-bearing CCR2-knockout mice (91). Further, neutrophils and macrophages are implicated in adaptive resistance to anti-angiogenic therapy in the Rip1Tag2 pancreatic neuroendocrine tumors. Therapeutic targeting of either population caused enhanced infiltration of the other myeloid cell population compensating for the loss of neutrophils and macrophages, respectively, which created an oscillating pattern of distinct immune-cell populations to facilitate adequate neovascularization (87).

Finally, innate lymphoid cells (ILCs) represent a recently identified heterogeneous family of mononuclear hematopoietic cells. Based on their lymphoid morphology, surface antigens, transcription factor expression, and cytokine productions (TH1, TH2, and TH17-like), ILCs have been classified into three major groups, termed ILC1, ILC2, and ILC3 (96). ILC3s elicit tumorigenic and angiogenic properties in part by secreting IL-17 (79, 97, 98). Notably, a subset of ILC1s share features with Natural killer (NK) cells, which are bone marrow-derived large granular effector lymphocytes. Cancer infiltrating NK cells have been shown to release angiogenic factors and immunosuppressive cytokines like VEGF, PlGF, and IL-8, similar to proangiogenic NK cells found in the developing endometrium (99). CD56^+^CD16^−^ NK cells from peripheral blood of patients with non-small cell lung cancer (NSCLC), especially squamous cell carcinoma (SCC) subtype, produce higher levels of VEGF, IL-8, and PlGF than those from healthy donors (100)

## Adaptive Immune Cells Regulate Angiogenesis

While adaptive immune cells are predominantly associated with immune surveillance, there is increasing evidence that they also regulate angiogenesis, although their exact functions in this process are just beginning to be revealed. In tumors, T-cells, due to their heterogeneous nature, appear to negatively or positively regulate tumor angiogenesis. Conditioned medium from Th2 and Th17 T-cells contained factors that enhanced angiogenesis *in vitro* in an endothelial sprouting assay and in a murine model of ischemia when released from an injectable alginate biomaterial. In contrast, Th1 conditioned medium induced regression of vascular tubes *in vitro* and was inefficient to instigate angiogenesis *in vivo* (101). In several mouse tumor model systems, CD8^+^ T-cells and CD4^+^ T-helper 1 cells have been shown to secrete IFNγ, which blocks vascular growth and triggers TAMs and TANs to produce the angiostatic chemokines CXCL 9,10, and 11 (3, 102, 103). In contrast, Treg cells suppress INFγ -expressing CD4^+^ Th1 cells and secrete VEGF via hypoxia-induced CCL28, that both promote an angiogenic tumor environment (104). The importance of VEGF production by T-cells was recently underscored by the finding that genetic deletion of VEGF in CD8^+^ T-cells enhanced tumorigenesis while it also exhibited hallmarks of tumor vessel normalization, with typical features of increased pericyte coverage of tumor blood vessels and decreased vessel tortuosity (105). Interestingly, the overall level of hypoxia was decreased consistently with better perfusion, a phenotype that was also observed when VEGF was deleted in TAMs (53). The lower numbers of infiltrating T-cells in tumors of VEGF mutant mice suggests that VEGF secreted by CD8^+^ T cells may affect T cell homing through the endothelial barrier and thus, its lack may be in part responsible for the augmented tumor growth (105). In support of these observations, human breast cancer tissues revealed an inverse correlation between VEGF levels and CD8^+^ T cell infiltration, and congruently linked T cell infiltration with the stage of vascularization (105). In further support, depletion of intratumoral CD4^+^ and CD8^+^ T-cell in mouse tumor models generated a more dysfunctional tumor vasculature with an increase in hypoxic areas. These effects could be reverted by CD8 influx and activity through checkpoint immunotherapy (anti-PD1 and/or anti-CTLA4), or by adoptive TH1 transfer, both invigorating tumor vessel normalization and reducing hypoxia (106). While these data provide evidence of T-cells in regulating vascular properties, the implication of B-cells remains somewhat elusive. Analysis of the overall B-cell population in tumors revealed that B-cells can secrete proangiogenic factors such as VEGF, FGF2, and MMP-9 and that they are able to promote immunosuppressive and proangiogenic properties in macrophages in an IgG-dependent manner (107, 108).

## Healing Tumor Wounds

The studies described above support the proposition that tumors generate a cytokine and chemokine milieu that stimulates an immunosuppressive and angiogenic environment displaying characteristics of the proliferative resolution phase in the wound repair process. Among the multifarious participants in this “wound scenario” are immune cells and blood vessels, which are functionally interconnected by mediators and molecules that commonly regulate both immunity and angiogenesis. Strategies to impede neovascularization were first developed with the intention to restrain tumor growth and “starve a tumor to death” (109). Antiangiogenic therapy targeting the VEGF-VEGFR and/or Ang-Tie2 pathway, however, has so far only provided beneficial effects in a subset of patients eliciting progression-free but not overall survival (77, 110, 111) because tumors find alternative strategies to adapt to the restrictions of vascular growth and reinstate growth (112). A major resistance mechanism is prompted by treatment-induced hypoxia and relies on recruiting distinct innate immune cells from the bone marrow to the tumor where they stimulate vascular growth in a VEGF-independent manner (5, 57, 59, 77). Importantly, the seminal observation of “vessel normalization” in responding tumors that pruned tumor vessels exhibited a more functional morphology with proper pericyte alignment improving blood flow and oxygenation also revealed a more immunostimulating environment with enhanced CD8 T cell influx (113, 114). Congruent with these studies, angiokinase inhibitors and anti-VEGFR antibodies facilitating vessel normalization in responding Rip1Tag2 PNET tumors converted intratumoral myeloid cells to an angiostatic and immunostimulating phenotype which was associated with an enhanced influx of cytotoxic CD8 cells (87). Due to continuous vessel pruning, however, hypoxic areas formed, leading to enhanced influx as well as proangiogenic and immunosuppressive polarization of innate immune cells concomitant with a drop of intratumoral CD8 cells. Mechanistically, CXCL12 and IL6 induction activated PI3Kγ signaling in intratumoral macrophages, neutrophils and MDSCs rendering them proangiogenic and immunosuppressive. PI3K-activated myeloid cells negated the antiangiogenic blockade and promoted tumor relapse (87). Further support stems from the observation that myeloid PI3Kγ signaling inhibits NFκB while it promotes C/EBPβ activation, thereby inducing a transcriptional program that favors immunosuppression (115). Importantly, therapeutic inhibition of myeloid PI3Kγ/δ was able to sustain the efficacy of antiangiogenic therapy. It polarized all myeloid cells to an angiostatic and immunostimulatory phenotype and enhanced CD8 T cell infiltration and activity in tumors (87). Tumors relapsing from antiangiogenic therapy did not only convert myeloid cells into a Th2 state, but they also enhanced the levels of the negative immune checkpoint regulator PD-L1 in tumor and stromal cells (116, 117). This displayed another mechanism of escaping immune surveillance because PD-L1 binds PD-1 on the surface of activated T-cells and thus blocks T-cell activity. Similarly to antiangiogenic therapy combined with a myeloid PI3K inhibitor, combined antiangiogenic (either anti-VEGF or anti-VEGF/Ang2 antibodies) and anti-PD-L1 immunotherapy had superior beneficial effects than respective monotherapies because the immunostimulating therapy blocked evasion from antiangiogenic therapy, while antiangiogenic-induced vascular normalization enhanced cytotoxic T cell infiltration and activation (116, 117). Notably, successful antiangiogenic immunotherapy could not only normalize tumor vessels but also generate high-endothelial venule (HEV)-like structures in some tumors that further enhanced lymphocyte infiltration to eradicate tumor cells (117). Another example demonstrating the benefits of antiangiogenic immunotherapy was demonstrated with the combination of the angiokinase inhibitor axitinib and anti-CTLA4 treatment. The drug combination provided extensive survival benefits in a mouse model of melanoma because it increased effector T-cell influx and dendritic cell maturation, and it reduced intratumoral MDSCs while the monotherapies failed (118). These observations resemble only a few examples for the support of targeting both the vascular and immune cell compartment to elicit enduring effects. Besides immune checkpoint inhibitors, there are certainly a variety of different drugs that have been developed for targeting signaling pathways in myeloid cells, including the inhibition of CSF1R, CXCR4, PI3Kγ/δ, CD47/SIRPα, and CCL2/CCR2 as well as the activation of CD40 and TLR7/9 (2, 119, 120) that could be combined with antiangiogenic therapies. From a mechanistic point of view, these results reveal a communality, i.e., the attempt to transit tumors from their proangiogenic and immunosuppressive phase into an immunostimulatory and angiostatic state similar to those phases observed during wound repair (Figures 1, 2). However, while the wound repair program transitions from an inflammatory stage to a proliferative resolution phase in order to properly close the wound, antiangiogenic immunotherapy in tumors attempts to do the opposite by awakening an inflammatory status in the “tumor wound” to abrogate tumor cells and invigorate tissue homeostasis.

## Conclusions

Ongoing clinical trials that combine antiangiogenic agents and immunotherapies like ICB or those targeting and modulating innate immune cells as well as strategies to directly enhance infiltration and activation of CD8 T-cells validate the concept of enhancing an immunostimulating environment in cancer. For example, several clinical trials are currently evaluating combined VEGF/VEGFR and PD-1/PD-L1 inhibitors for various cancer types including renal cell carcinoma, recurrent glioblastoma, ovarian cancer and colorectal cancer (NCT03024437, NCT02659384, NCT02873962, NCT02017717). The clinical trial IMmotion150 (NCT01984242) in patients with naïve renal cell cancer (mRCC) assessed the combination of anti-PD-L1 (atezolizumab) with or without bevacizumab, against the standard-of-care angiokinase inhibitor, sunitinib (121). Combining anti-PD-L1 with bevacizumab was more efficacious than sunitinib in patients with PDL1-positive tumors. Interestingly, the mutational rate and neoantigen burden of tumors did not correlate with progression-free survival (PFS), but angiogenesis and myeloid inflammatory gene expression signatures associated strongly with PFS within and across the treatments arguing that these signatures could be utilized as prospective biomarkers (121, 122). Similar to the results obtained in preclinical tumor models described above, myeloid-driven inflammation in tumors appeared to be a resistance mechanism to anti-PD-L1 monotherapy in mRCC which could be overturned by bevacizumab (87, 116, 117). These first clinical results are certainly promising and together with upcoming clinical trials, will be able to thoroughly assess the effectiveness of antiangiogenic immunotherapies in improving and enduring survival of cancer patients.

## Author Contributions

All authors listed have made a substantial, direct and intellectual contribution to the work, and approved it for publication.

### Conflict of Interest Statement

The authors declare that the research was conducted in the absence of any commercial or financial relationships that could be construed as a potential conflict of interest.
